# A Novel Replication-Competent Vaccinia Vector MVTT Is Superior to MVA for Inducing High Levels of Neutralizing Antibody via Mucosal Vaccination

**DOI:** 10.1371/journal.pone.0004180

**Published:** 2009-01-13

**Authors:** Xiaoxing Huang, Bin Lu, Wenbo Yu, Qing Fang, Li Liu, Ke Zhuang, Tingting Shen, Haibo Wang, Po Tian, Linqi Zhang, Zhiwei Chen

**Affiliations:** 1 Modern Virology Research Center and AIDS Center, State Key Laboratory of Virology, College of Life Sciences, Wuhan University, Hubei, People's Republic of China; 2 AIDS Institute, Li Ka Shing Faculty of Medicine, The University of Hong Kong, Hong Kong SAR, People's Republic of China; 3 AIDS Research Center, Institute of Pathogen Biology, Chinese Academy of Medical Sciences, Beijing, People's Republic of China; 4 Comprehensive AIDS Research Center, Tsinghua University, Beijing, People's Republic of China; Beijing Institute of Infectious Diseases, China

## Abstract

Mucosal vaccination offers great advantage for inducing protective immune response to prevent viral transmission and dissemination. Here, we report our findings of a head-to-head comparison of two viral vectors modified vaccinia Ankara (MVA) and a novel replication-competent modified vaccinia Tian Tan (MVTT) for inducing neutralizing antibodies (Nabs) via intramuscular and mucosal vaccinations in mice. MVTT is an attenuated variant of the wild-type VTT, which was historically used as a smallpox vaccine for millions of Chinese people. The spike glycoprotein (S) of SARS-CoV was used as the test antigen after the S gene was constructed in the identical genomic location of two vectors to generate vaccine candidates MVTT-S and MVA-S. Using identical doses, MVTT-S induced lower levels (∼2-3-fold) of anti- SARS-CoV neutralizing antibodies (Nabs) than MVA-S through intramuscular inoculation. MVTT-S, however, was capable of inducing consistently 20-to-100-fold higher levels of Nabs than MVA-S when inoculated via either intranasal or intraoral routes. These levels of MVTT-S-induced Nab responses were substantially (∼10-fold) higher than that induced via the intramuscular route in the same experiments. Moreover, pre-exposure to the wild-type VTT via intranasal or intraoral route impaired the Nab response via the same routes of MVTT-S vaccination probably due to the pre-existing anti-VTT Nab response. The efficacy of intranasal or intraoral vaccination, however, was still 20-to-50-fold better than intramuscular inoculation despite the subcutaneous pre-exposure to wild-type VTT. Our data have implications for people who maintain low levels of anti-VTT Nabs after historical smallpox vaccination. MVTT is therefore an attractive live viral vector for mucosal vaccination.

## Introduction

Vaccinia virus (VV) provided excellent prophylactic immunity to variola virus, the causative agent of smallpox, and led to the eradication of this fatal disease in the world [1]–[3]. In recent years, VV has also been successfully used as a live vaccine vector for the prevention or eradication of other infectious diseases [4], [5] because of its advantage for delivering the expression of foreign antigens in eukaryotic cells [6]–[8]. A considerable number of different strains of VV have been adapted to serve as vaccine vectors such as NYVAC, NYCBOH, MVA and Tian Tan [6], [9]–[12]. These VV strains have been engineered to express antigens of herpes simplex virus, hepatitis B virus, rabies virus, influenza virus, human immunodeficiency virus (HIV), respiratory syncytial virus (RSV), severe acute respiratory syndrome coronavirus (SARS-CoV) and other pathogens, respectively [13]–[21]. Among them the modified vaccinia Ankara (MVA) has probably been the most widely studied vaccinia vector especially due to its excellent safety profile in humans [17]–[19], [21], [22]. MVA vaccine elicited levels of cytotoxic T lymphocyte (CTL) responses that were comparable to those induced by replication-competent VV strains [23], [24]. Importantly, vaccination with MVA protected macaques against pathogenic monkeypox challenge [23] and MVA-based recombinant vaccines were able to induce protective immune responses against different viruses including SARS-CoV, influenza virus and RSV [14], [15], [23], [25], [26]. The immunogenicity of MVA expressing HIV antigens, however, was not satisfactory as described in recent human clinical trials [27], [28]. Moreover, since MVA requires large clinical doses (10^8^ PFU or higher) and its propagation needs special pathogen free (SPF) primary chicken embryo fibroblast (CEF) cells, it has been a manufacture burden to produce a sufficient quantity of clinical grade products especially in developing countries [29], [30]. It is therefore necessary to study other vaccinia-based vaccine vectors.

Some studies have been carried out to investigate whether or not different vaccinia vectors would offer any advantages especially for inducing protective immune responses [31]–[33]. This is a critical issue because different VV vectors may harbor distinct profiles in terms of immune modulation and host virulence [34],[35]–[37]. Furthermore, studying mucosal vaccination is also critical because the major mode of transmission for many viruses including HIV, SARS-CoV, influenza virus, etc., was through mucosal surfaces. Conventional replication-competent vaccinia vectors are considered to be effective in mucosal vaccination but their safety issues may limit their widespread use in humans [32]. It is, therefore, suggested that attenuated replication-competent vaccinia vectors should be further studied for mucosal vaccination. Till now, it remains unknown whether or not our newly developed replication-competent modified vaccinia Tian Tan (MVTT) would offer any advantage over the non-replicating MVA for mucosal vaccination after a test antigen is constructed under an identical promoter in the same genomic location of two live vectors, respectively.

Vaccinia Tian Tan (VTT) was historically used as a vaccine for millions of Chinese people during the worldwide smallpox prevention campaign, which led to the variola eradication in China before 1980 [38]–[40]. Similar to other vaccinia strains, VTT is a member of the orthopoxvirus genus. Although there is no historical report on the safety profile of VTT in humans, it has been suggested that VTT likely exhibits good immunogenicity, moderate reactogenicity, and relatively mild complications [11], [12]. A recent study suggested that the intranasal delivery of a VTT-based vaccine, when used in combination with a DNA, induced strong HIV-specific immune responses [20]. The safety profile of this particular vaccine for mucosal use was not mentioned in the publication. We however demonstrated that the unmodified VTT retains its virulence and pathogencity in animal studies especially after intranasal administration [38]. Our data indicated that VTT should be modified to be an intranasal vaccination vector [38]. We have since generated modified vaccinia Tian Tan (MVTT) strains by attenuating the virulence of VTT through viral genomic engineering and clonal selection [11].

Since the Merck's CTL-oriented HIV vaccine has recently failed in clinical trials, it becomes desirable to identify an effective live vector for eliciting neutralizing antibodies (Nabs). Here, we studied the potential of our MVTT as a vaccine vector for inducing Nabs. Specifically, we report findings on a head-to-head comparison study between MVA and MVTT vectors for mucosal vaccination. The major uniqueness of MVTT is that it is an attenuated yet replication-competent strain as tested in mammalian cells. The spike glycoprotein (S) of SARS-CoV was used as the test antigen after its gene was constructed in the identical genomic location of two live vectors to generate vaccine candidates MVTT-S and MVA-S [14], [41]. By evaluating the specific neutralizing antibody (Nab) response against SARS-CoV, we determine the immunogenicity profiles of two distinct live vaccinia vector systems *in vivo*.

## Materials and Methods

### Virus and vector

**Figure 1:**
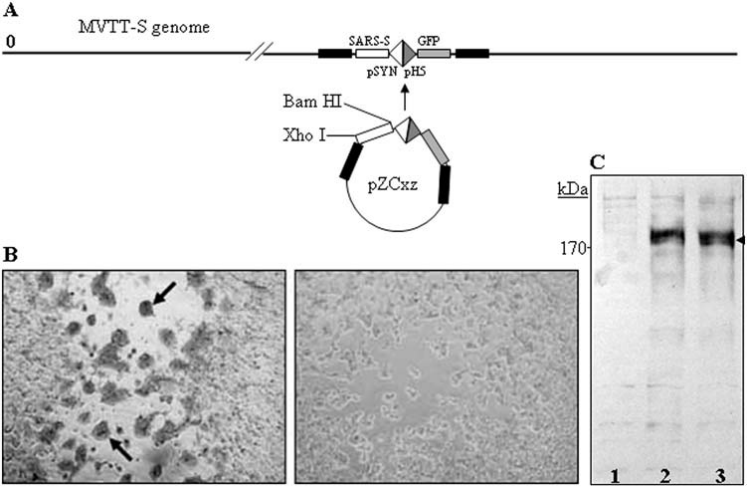
The schematic representation of MVTT-S construction and the expression of the S-glycoprotein in cells infected with MVTT-S. (A) The S gene of SARS-CoV was introduced, together with GFP gene, each under a separate promoter, into the genome of MVTT. The restriction enzymes Bam HI and Xho I were used for constructing MVTT-S. The insertion region corresponds to the Del III region of MVA. (B) The S-glycoprotein was detected on Vero cells infected with MVTT-S (left image) using a rabbit anti-S specific antibody in an immunohistochemical staining assay. No S-glycoprotein expression was detected on Vero cells infected with the wild-type VTT (right image). (C) The comparable level of S-glycoprotein expression was detected in CEF cells infected with 1×10^6^ PFU of either MVTT-S (lane 2) or MVA-S (lane 3) using the Western blot assay. Uninfected CEF was included as a negative control (lane 1).

The background of the parental vaccinia Tian Tan has been described in our previous publications [11], [38]. A modified dual-promoter insertion vector, pZC_xz_ was constructed to target the genomic region of VTT, which corresponds to the Del III region of MVA [14]. pZC_xz_ contains two promoters namely pSYN and pH 5, which are both vaccinia virus-specific early/later promoters. Using pZC_xz_, S gene and reporter green fluorescent protein (GFP) gene were incorporated into the genome of VTT to construct the recombinant vaccinia virus MVTT-S using a homologous recombination method in African green monkey kidney (Vero) cells (Fig. 1). The virus was purified through consecutive plaque selection using GFP as a surrogate marker under a fluorescence microscope. The stability of S in MVTT was determined by double-staining S protein and vaccinia specific antigens for 50 foci of the ninth passaged viral stock, which is similar to a technique we recently published [11]. Using similar techniques, MVA-S was constructed by inserting S gene into the Del III region of MVA genome as we previously described [14].

### Immunohistochemistry and Western blot assays

An immunohistochemistry assay was developed for the detection of SARS-CoV S glycoprotein expressed on cell surface. Briefly, Vero cells were grown in 35-mm culture dishes to reach 80% confluence and were subsequently infected with MVTT-S at a multiplicity of infection (m.o.i.) of 0.01. After viral absorption for 90 min, cells were washed three times with culture medium and then incubated at 37°C for another 24 hours (h) to 48 h before immunostaining. After the incubation, cells were fixed with a cold 1∶1 solution of methanol/acetone for five min and then incubated for 1 h at room temperature with a rabbit anti-S serum diluted (1∶80) in phosphate buffered saline (PBS) containing 2% fetal bovine serum (FBS). Cells were washed and then incubated for 1 h at room temperature with 1∶1500 diluted Protein-A conjugated to horseradish peroxidase (Zhongshan Biotech, Beijing, China). After wash, the color was developed by incubation for 30 min with the substrate solution consisting of 10 µl of 30% H_2_O_2_ and 0.2 ml of an ethanol saturated dianishidine (Sigma, St. Louis, MO, USA) solution in 10 ml of PBS [11], [38]. For Western blot analysis, cells were infected with vaccinia virus at a multiplicity of infection (MOI) of 0.1. 48 h after transfection, cells were lysed on ice for 30 minutes in 100 µL of lysis buffer (50 mM of Tris-HCl [pH 8.0]; 137 mM of NaCl; 2 mM of ethylenediaminetetraacetic acid [EDTA]; 0.5% NP-40; 10% glycerol; and 1 µg/mL each of pepstatin, leupeptin, and pefabloc), cleared of lysate (14,000 rpm for 10 minutes at 4°C), boiled at 100°C for 10 minutes, and run on 10% sodium dodecyl sulfate–polyacrylamide gel electrophoresis (Invitrogen, Carlsbad, CA, USA). After proteins were transferred to polyvinylidene difluoride membrane (Invitrogen), blots were blocked in 5% milk and 0.5% BSA in PBS; washed; and incubated with rabbit anti-S (1∶100 ratio) polyclonal antibodies. The blots were washed and incubated with 1∶4,000 diluted protein-G horseradish peroxidase (HRP) conjugate (Bio-Rad, Hercules, CA, USA). Immunofluorescence was measured with the enhanced chemoluminescence (ECL) plus kit (Amersham Biosciences, Piscataway, NJ, USA).

### Preparation of viral stocks and viral virulence test

VTT and MVTT-S viral stocks were propagated in Vero cells and then purified by centrifugation through a 36% sucrose cushion. MVA-S stock was prepared and purified using chicken embryo fibroblast (CEF) cells according to a procedure described previously [14]. Both viral stocks were titrated simultaneously in CEF by a plaque forming assay using crystal violet staining or counting the plaques with GFP expression.

To determine the viral virulence *in vivo*, two groups of six-week old mice were inoculated with 10^5^ and 10^6^ PFU of MVTT-S via the i.n. route, respectively. Each group had six mice. The viral virulence was subsequently determined by the daily measurement of animal body weight change for a period of 31 days.

### Immunization of animals

For dose escalation study, groups of three six-week-old female BALB/c mice were immunized by intramuscular (i.m.) injection of MVTT-S or MVA-S at weeks 0 and 3. Each group of mice was inoculated with one of 10^4^, 10^5^ and 10^6^ PFU of either viruses, or a saline control. To determine the best route of vaccination, a sub-optimal dose (10^5^ PFU) was used for each group of three BALB/c mice (6-week-old) at weeks 0 and 3. The vaccination routes included intranasal (i.n.), intraoral (i.o.), intrarectal (i.r.), intradermal (i.d.), subcutaneous (s.c.) and intraperitoneal (i.p.) inoculations. For mucosal vaccination study, groups of 3 BALB/c mice (6-week-old) were immunized with 10^5^ PFU of each virus at weeks 0 and 3 via i.m., i.n., i.o., i.r. routes. The vaccinated mice were sacrificed two weeks after the second injection and their blood samples were used for analysis. The animal experimental protocols were approved by institutional animal ethic committee and followed the national guidelines for the use of animals in scientific research.

### Pre-existing immunity to VTT

For the experiment of evaluating whether the pre-exposure to VTT would interfere with the immunogenicity of MVTT-S, groups of six-week-old BALB/c mice were immunized by the s.c. injection of 10^6^ PFU VTT at day 0. These mice were kept for six months and then were inoculated with 10^6^ PFU MVTT-S or MVA-S twice at one month interval via i.m.; s.c.; i.n.; i.o. routes, respectively. Sera were collected two weeks after each inoculation for analysis.

In parallel, as placebo controls, other groups of six-week-old BALB/c mice were immunized by the s.c. injection of PBS at day 0. These mice were kept for six months and then were inoculated with 10^6^ PFU MVTT-S or 10^6^ PFU MVA-S or PBS twice at one month interval via i.m.; s.c.; i.n.; i.o. routes, respectively. Sera were collected two weeks after each inoculation for analysis.

### Neutralization assay

A pseudovirus-based neutralization assay was established to determine the humoral immune responses against SARS-CoV [14]. The pseudotype virus was generated by co-transfecting 293T cells with two plasmids pcDNA-Sopt9 and pNL4-3Luc^+^Env^−^Vpr^−^ carrying the optimized S gene and a human immunodeficiency virus type 1 backbone, respectively, as we previously described [14]. The neutralizing activity of heat-inactivated sera (56°C, 30 min) was determined by mixing 10 ng of pseudotype virus (in 30 µl) with diluted serum (in 30 µl) at 37°C for 1 h. After neutralization, the mixture was combined with 16 ng polybrene (in 40 µl medium) and added to HEK293T-ACE2 cells (10,000 cells per well in 100 µl). Cells were washed with PBS and lysed (1× Cell Culture Lysis Reagent; Promega, Madison, WI, USA) 56–72 h after infection. Luciferase activity was measured and the percentage of neutralization was calculated. The assay for vaccinia neutralization was based on a previous publication using the fluorescence-activated cell sorting (FACS) analysis [42]. For this assay, a VTT-based recombinant virus was constructed to express the green fluoresces protein (GFP) gene.

## Results

### Design and characterization of a recombinant MVTT that expresses the SARS-CoV spike glycoprotein

We have previously generated a MVA-based vaccine, MVA-S, which was able to prevent SARS-CoV infection in Chinese rhesus monkeys [14]. In order to make a reasonable comparison between MVA and MVTT as live vaccine vectors, we used the same strategy for the construction of MVA-S to generate a MVTT-based vaccine. A modified shuttle vector pZC_xz_ was constructed to target the S gene into the MVTT genome in a location which corresponds to the Del III region of MVA-S (Fig. 1A). This shuttle vector contains dual promoters which allow the simultaneous expression of two target genes. Within this vector, the S gene of SARS-CoV was constructed under the strong synthetic promoter pSYN, whereas a reporter GFP gene was under a separate relatively weaker promoter pH 5 (Fig. 1A). Since both genes were included within the same insertion frame, the GFP served as a surrogate marker for the selection of recombinant VTT carrying the S gene. Using this technique, we were able to generate and to purify the recombinant virus MVTT-S. The positive plaque was selected under a fluorescence microscope and subsequently confirmed by an immunohistochemical assay using an S glycoprotein specific antibody (Fig. 1B, left image). Moreover, the S protein was also detected by a rabbit antibody bound to the N-terminal 400 amino acids of the protein in a Western blot analysis. The S protein appeared at the position above 170 kDa, which was consistent to our previous observation (Fig. 1C) [14]. Comparable of levels of S-glycoprotein expression were detected in CEF cells using an identical dose of viral inoculation (Fig. 1C). In addition, MVTT-S was passaged on Vero cells nine times. By evaluating 50 foci after the 9^th^ passage, we found that 100% of the foci expressed both S glycoprotein and vaccinia proteins. These passages, therefore, did not lead to the loss of S glycoprotein expression, indicating that MVTT-S is likely genetically stable.

### Attenuated phenotype of MVTT-S in mice

**Figure 2:**
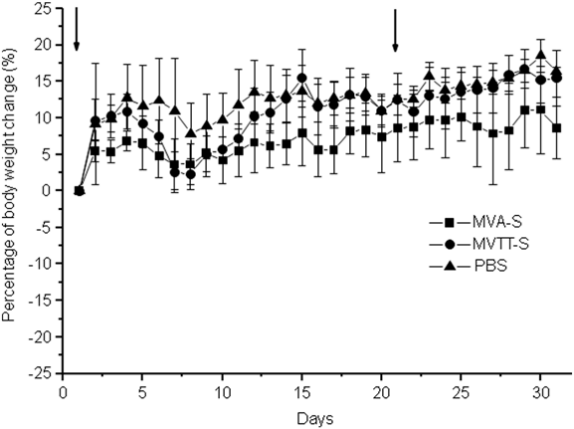
Virulence of MVTT-S in mice after i.n. inoculation. Groups of six mice were inoculated with MVTT-S, MVA-S or PBS twice at a dose of 10^6^ PFU at a three-week interval. The body weight of animals was measured overtime post inoculation. The average values (±standard error) of body weight are plotted for each group of animals. The arrow indicates the time of each viral inoculation.

Considering that the insertion of foreign gene products might alter the virulence of MVTT, we have evaluated the *in vivo* toxicity of MVTT-S in animals. The inbred BALB/c mouse was chosen for the assessment of MVTT-S virulence as we previously described [11], [38]. The data collected from the 10^6^ PFU group were presented in Figure 2. Two independent experiments were conducted with consistent results obtained. None of the mice died during the experiment period. Furthermore, mice infected with MVTT-S did not show signs of weight loss (Fig. 2). The body weight change in mice infected with MVTT-S was comparable with the control mice. These results suggest that MVTT-S likely retains an attenuated phenotype and is likely safe for intranasal vaccination.

### MVA-S induces slightly higher levels of Nab response than MVTT-S via the i.m. route of inoculation

**Figure 3:**
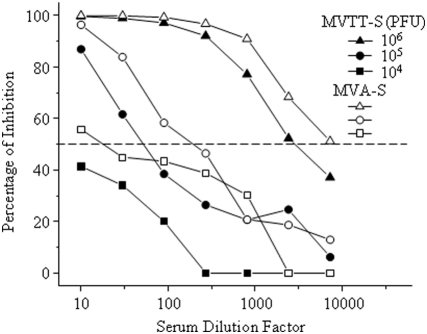
Anti-S-specific Nab response in mice vaccinated with either MVTT-S or MVA-S via the i.m. route. Groups of 3 mice were injected with MVTT-S (solid symbol) or MVA-S (empty symbol) twice at a dose of 10^4^ (square), 10^5^ (cycle), 10^6^ (triangle) PFU at a three-week interval. Sera were collected two weeks after each vaccination. A pseudotype-based neutralization assay was performed to determine the level of Nab response. The Y axis stands for the percentage of inhibition whereas the X axis represents the serum dilution factor. The dash line indicates the level of 50% of viral inhibition. The neutralization experiment was repeated twice with similar results obtained. Moreover, two independent immunization experiments were conducted with consistent results obtained.

As described previously, a pseudotype-based neutralization assay was established to characterize the immune sera generated in mice [14]. The major advantage of this assay is the elimination of using live SARS-CoV in the traditional neutralization assay. We used this assay to measure the serum neutralizing activity in animals immunized with MVA-S and MVTT-S. A total of six groups of mice were immunized with either MVA-S or MVTT-S, respectively, through i.m. inoculation. Three mice as one group were given 10^4^, 10^5^, or 10^6^ PFU of each vaccine. All of the animals were immunized twice at a three-week interval. Serum samples were collected and subjected to the neutralization assay two weeks after the second immunization. As controls, three additional mice received placebo. As depicted in Fig. 3, since the difference was about two to three-fold based IC_50_ values (the 50% inhibitory concentration), it is likely that MVA-S induced slightly higher level of Nabs than MVTT-S via i.m. inoculation under the experimental condition. Two independent immunization experiments were conducted with similar results obtained. Moreover, there was a clear dose dependency among three MVA-S or MVTT-S dosing groups. The IC_50_ values reached over 1∶5,000 in the high-dose groups. In contrast, the groups of control mice did not yield any Nabs.

### MVTT-S induced at least over 100-fold higher Nab response than MVA-S via i.n. or i.o. inoculation

**Figure 4:**
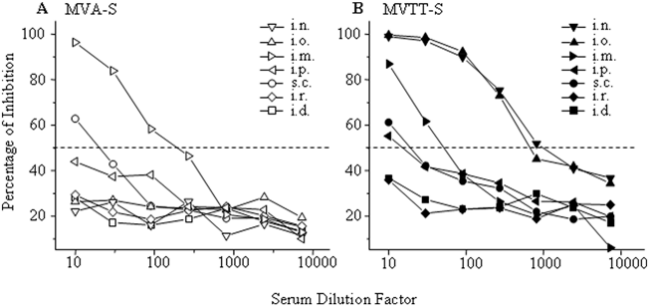
Anti-S-specific Nab response in mice vaccinated with MVA-S (A) or MVTT-S (B) via various routes. Groups of 3 mice were inoculated with 10^5^ PFU MVTT-S or MVA-S twice at a three-week interval via seven different routes including i.m., i.d., s.c., i.o., i.n., i.r. and i.p. The sera were collected two weeks after the last injection and subjected to the neutralization assay. The Y axis stands for the percentage of inhibition while the X axis represents the serum dilution factor. The dash line indicates the level of 50% of viral inhibition. The experiment was repeated twice with similar results obtained.

To explore the potential use of MVA-S or MVTT-S as a mucosal vaccine vector, we further tested these two vaccines in mice. Seven groups of three BALB/c mice (6-week-old) were immunized via i.n., i.o., i.m., i.r., i.d., i.p., s.c. routes using an identical sub-optimal dose (10^5^ PFU), respectively. We chose the sub-optimal dose in order to avoid the plateau effort of immune responses under high doses of inoculation in mice [21]. Similar to i.m. inoculation, all of the animals were immunized twice at a three-week interval. Serum samples were collected, pooled by equal volume and subsequently subjected to the neutralization assay two weeks after the second immunization. As controls, three mice received placebo. As shown in Fig. 4A, no significant levels of Nabs were induced in mice that were immunized with MVA-S except for i.m. and s.c. routes. Less than fifty percent of the virus was neutralized after the sera were diluted by 1∶10 for the rest groups. In particular, i.n. or i.o. immunization only induced antibodies capable of neutralizing about 25–30% of virus at 1∶10 dilution. In contrast, MVTT-S induced much higher levels of SARS-CoV-specific Nabs in mice that were immunized with MVTT-S via the i.n. or i.o. routes of inoculations (Fig. 4B). Fifty percent of the virus was neutralized after the sera were diluted by 1∶900, which is at least 100-fold better than that of MVA-S (Fig. 4A). These levels of response based on IC_50_ were approximately 10-fold higher than that based on i.m. vaccination when the same dose of inoculum was used (Fig. 4B). Therefore, MVTT-S is superior to MVA-S once delivered via i.n. or i.o. routes. During the experiments, the placebo groups of control mice did not yield any detectable Nabs.

### Pre-mucosal exposure to wild-type VTT prevents the subsequent Nab response using the same route of vaccination

**Figure 5:**
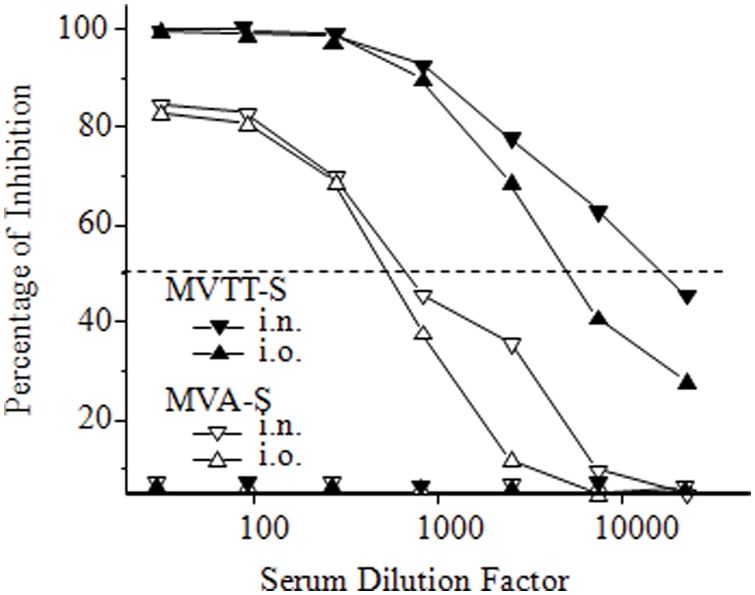
Influence of pre–VTT vaccination on the efficacy of MVTT-S or MVA-S via the same i.n. or i.o. route of immunization, respectively. Groups of 4 mice were inoculated with 10^6^ PFU VTT via i.n. or i.o. route, and were subsequently (six months later) injected with 2×10^6^ PFU MVTT-S or MVA-S twice at one month interval via the same routes. No anti-S Nab responses were detected in these animals (flat lines on the X axis). In contrast, placebo mice, that were previously given PBS, developed significant levels of anti-S Nab responses after received two MVTT-S (solid symbol) or MVA-S (empty symbol) injections via the same i.n. or i.o. route. The Y axis stands for the percentage of inhibition whereas the X axis represents the serum dilution factor. The dash line indicates the level of 50% of viral inhibition. The experiment was repeated twice with similar results obtained.

Considering that pre-existing immunity would interfere with the effectiveness of live viral vector-based vaccines, we sought to determine whether this would be the case for MVTT-S and MVA-S vaccines. To address this issue properly, four groups of four mice were inoculated with 10^6^ PFU VTT via i.n. and i.o. routes, respectively. Another four groups of mice received placebo. After six months of resting, the animals were vaccinated twice with 2×10^6^ PFU MVTT-S or MVA-S at a month interval via the autologous route, and subsequently subjected to the neutralization assay two weeks after the second immunization. We found that the pre-mucosal exposure to wild-type VTT had a profound effect on the effectiveness of MVTT-S and MVA-S vaccines. No neutralizing antibody response was detected among the mice that had previously been exposed to wild-type VTT at the serum dilution of 1∶10. Of note, 2×10^6^ PFU is the highest dose possible for mucosal vaccination based on the concentration of our viral stocks. Based on IC_50_, placebo mice inoculated with MVTT-S developed 10-fold (i.o.) to 50-fold (i.n.) higher levels of Nab responses than those received MVA-S (Fig. 5). Apparently, more than 20-fold of MVA-S (2×10^6^ PFU in Fig. 5) is required to induce similar levels of Nabs that were elicited by MVTT-S (10^5^ PFU in Fig. 4B) via either i.n. or i.o. inoculation.

### Mucosal vaccination overcomes the pre-existing immune response induced by the s.c. exposure to wild-type VTT

**Figure 6:**
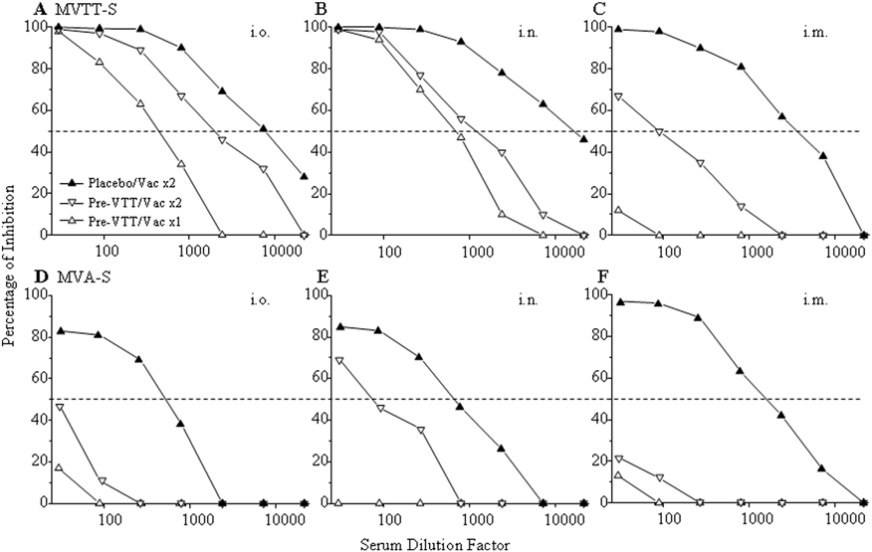
Influence of the s.c. pre-VTT vaccination on the efficacy of MVTT-S (top panel) or MVA-S (bottom panel) via heterologous route of immunization. Experimental groups of 4 mice (empty symbol) were inoculated with 10^6^ PFU VTT via the s.c. route, and were subsequently (six months later) given one (Vac×1) or two (Vac×2) injections of 2×10^6^ PFU MVTT-S or MVA-S twice at one month interval via heterologous routes including i.o. (A or D), i.n. (B or E) and i.m. (C or F), respectively. Placebo mice (solid symbol), that were previously given PBS, received two injections of 2×10^6^ PFU MVTT-S or MVA-S at one month interval via the same routes accordingly. Sera were collected two weeks post the first and the second inoculation and subjected to the neutralization assay. The Y axis stands for the percentage of inhibition while the X axis represents the serum dilution factor. The dash line indicates the level of 50% of viral inhibition. The experiment was repeated twice with similar results obtained.

Due to the suppression of MVTT-S in the previous experiment as shown in Fig 5, we sought to investigate the situation that mimics the human smallpox vaccination. In this case, six experimental groups of four mice were pre-inoculated with 10^6^ PFU VTT via the s.c. route. Another three groups of mice received placebo. After six months of resting, one of each experimental and control groups of animals were vaccinated twice with 2×10^6^ PFU MVA-S or MVTT-S at a month interval via i.n., i.o. and i.m. routes, respectively. The serum samples were subsequently subjected to the neutralization assay two weeks after the second immunization. Interestingly, the s.c. pre-exposure to VTT had much less interference on the immunogenicity of MVTT-S when delivered using a heterologous route. After the s.c. pre-exposure to VTT, i.o. (Fig. 6A) and i.n. (Fig. 6B) were still able to induce higher levels (20-to-50-fold) of anti-S Nab response when compared with the i.m. immunization after the second mucosal vaccination (Fig. 6C). Some reduced responses, however, were observed for i.o. (∼4-fold, Fig. 5A) and i.n. (∼10-fold, Fig 5B) when compared with animals without pre-exposure to VTT. In contrast, the s.c. pre-exposure to VTT had a profound negative effect on subsequent MVA-S mucosal vaccinations. Only two i.n. vaccinations induced some Nabs (Fig. 6E, IC_50_ = 1∶80).

### The pre-existing level of anti-VTT Nabs probably affects the effectiveness of MVA-S and MVTT-S

Considering that MVTT-S induced variable levels of anti-S Nab when different routes of inoculation were used (Fig. 3), we sought to determine whether similar effects apply to anti-VTT vector immune response. To address this issue, we measured the level of anti-VTT Nabs in mice after three weeks and six months of VTT vaccination using the FACS-based neutralization assay. Based on the values of IC_50_, it was obvious that higher levels of anti-VTT Nabs were induced via i.n. (1∶1384) and i.o. (1∶675) than via s.c. (1∶68) inoculations just before the administration of MVA-S and MVTT-S (Table 1). This finding suggests that these higher levels of anti-VTT Nabs probably affect the effectiveness of the subsequent MVA-S and MVTT-S vaccination (Fig. 5). Interestingly, the average titer of 1∶68 among subcutaneously vaccinated mice had much less effects on the subsequent i.o. or i.n. MVTT-S vaccination (Fig. 6A and 6B) when compared with i.m. route (Fig. 6C). Similar findings were obtained with MVA-S (Fig. 6D, 6E and 6F). In addition, we determined the anti-S Nab response in the presence of pre-existing anti-VTT Nabs three weeks after the first immunization of MVA-S and MVTT-S. We found that i.n. and i.o. inoculations of MVTT-S were able to induce anti-S Nab responses (Fig. 6A and 6B). These responses were further enhanced after the second inoculation of MVTT-S via i.o. (∼6-fold, Fig. 6A) or i.n. (∼2-fold, Fig. 6B) routes. The second immunization also significantly boosted anti-S Nab responses against MVTT-S via i.m. (Fig. 6C) or MVA-S via i.n. (Fig. 6E) inoculations, respectively. Therefore, the second MVTT-S immunization is necessary for boosting Nab response in the presence of the pre-existing anti-VTT immunity.

**Table 1:** The titer of anti-VTT neutralizing antibody (IC_50_).

Route	Day 21*	Day180
s.c.	<20	1∶68
i.n.	1∶540	1∶1384
i.o.	1∶500	1∶675

* indicates the date of sampling after various routes of vaccination.

IC_50_ stands for the antibody titer when 50% of virus was neutralized.

## Discussion

MVTT offers greater advantage than MVA for inducing high level of systemic Nab response against SARS-CoV through mucosal routes of vaccination. Considering that the induction of Nab is one of the key elements for a successful vaccine, we chose to use the spike glycoprotein (S) of SARS coronavirus as a test antigen to understand the immunogenicity profiles of MVA and MVTT. Another reason to choose S glycoprotein is that we have previously demonstrated that MVA-S induces protective Nabs that contributed to the protection of pathogenic SARS-CoV infection in Chinese macaques [14]. To make a fair comparison, the S gene of SARS-CoV was constructed in the equivalent genomic location of two live vectors (Fig. 1). We found that MVA-S induced 2-3-fold higher levels of Nab response than MVTT-S in mice via the i.m. route of inoculation using sub-optimal doses (Fig. 3). This finding suggests that it is possible that the replicating MVTT-S does not seem to offer much advantage when delivered intramuscularly, a systemic way of vaccination. We noticed that the advantage for MVA-S i.m. immunization reduced when a higher dose of vaccine was tested (Fig. 6C and 6F). This observation suggests that MVA-S has probably reached the plateau of Nab response given its non-replicating nature. Another possibility is related to the experimental setting because there was a long resting period (six months) after the animals received the placebo inoculation (Fig. 6). We, however, found that the i.n. or i.o. inoculation of MVTT-S induced the highest levels of Nab response which was over at least 100-fold higher than those of MVA-S using a sub-optimal dose (10^5^ PFU) (Fig. 4). Moreover, we demonstrated that a minimum of 20-fold or more of MVA-S (2×10^6^ PFU in Fig. 5) is required in order to induce similar levels of Nabs that were elicited by 10^5^ PFU of MVTT-S (Fig. 4B) via either i.n. or i.o. inoculation. These findings are new and suggest that a much higher minimal dose is required for MVA-S to be immunogenic via mucosal vaccination. Similar results have not been reported by comparing other replicating vaccinia strains with MVA for inducing Nab responses [9], [43]. Since these levels of Nab response are 10-fold higher than those induced via the i.m. route, our findings suggest that MVTT-S is superior to MVA-S when delivered via the i.n. or i.o. route. There are some possibilities to explain our findings. First, MVTT-S may target the mucosa-associated antigen presenting cells more efficiently. Second, the replication competency of MVTT-S may have growth advantage in mucosal tissues in providing continuous immune stimulation. Third, MVTT-S may contain mucosa-specific immune regulatory proteins which MVA-S does not have. Further studies will be needed to determine the underlined mechanism of MVTT-S-mediated mucosal vaccination. Our data provide direct evidence that the use of different vaccinia vectors may produce significantly different immunogenicity profiles for an identical test antigen. Our MVTT system may potentially assist some weak viral immunogens (e.g HIV-1 gp140) for inducing better Nab responses [21]. To this end, the intranasal delivery of a VTT-based vaccine, when used in combination with a DNA, induced strong HIV-1 specific immune responses in a recent small animal study [20]. Since VTT strains may come from different sources and display distinct *in vivo* toxicity and immunogenicity profiles, vectors aiming for clinical use should be carefully studied [11].

Mucosal vaccination overcomes the pre-existing immunity that was induced through s.c. route of vaccination by wild-type VTT. Pre-existing immunity is often the issue which is related to the effectiveness of live viral vector-based vaccines [37], [44]. It is also a critical issue for the clinical development of a vaccinia-based vaccine. Consistent with previous findings on other vaccinia-based vectors, we found that pre-mucosal exposure to wild-type VTT had a profound effect in preventing the subsequent Nab response using the same route of vaccination by MVTT-S (Fig. 5). In contrast, if the pre-exposure to wild-type VTT was through the s.c. route, mucosal vaccination via either i.n. or i.o. inoculation was able to overcome the pre-existing immune response to the vector, and to induce high level of anti-S Nabs (Fig. 6A and 6B). Since the latter situation mimics the conventional smallpox vaccination, our data have critical implications for millions of people who had been historically vaccinated with VTT through skin scarification. In support of this notion, we found that most of VTT-vaccinated Chinese people only maintain low levels of Nabs (<1∶60, data not shown). Mucosal vaccination, as a non-invasive or a needless procedure, has great implications for the large scale of vaccination programs in developing countries especially for nations with huge populations. In addition, the replication competent nature of MVTT permits a minimized dose of each vaccination and reduces the manufacture burden, which makes MVTT-based vaccine become cost effective. MVTT, therefore, is an attractive attenuated replicating-competent vaccinia vector for mucosal vaccination. Other approaches however for assisting mucosal delivery of MVTT-based vaccine should be further studied to overcome the anti-vector pre-existing immunity [44].

Mucosal vaccination may offer great advantage for inducing protective immune responses at the mucosal sites of viral transmission. Consistent with the anti-S Nab response induced by MVTT-S, the i.n. or i.o. inoculation of wt-VTT also induced much higher levels of Nab response against the viral vector than that of the s.c. vaccination (Table 1). In fact, this anti-VTT response probably played a critical role in eliminating the subsequent inoculation of MVTT-S through the autologous route or in preventing viral dissemination, and therefore prevented the induction of Nab response against S glycoprotein. On one hand, the data suggest that both VTT and MVTT probably offer great advantage for inducing protective Nab responses at the mucosal sites of viral transmission. On the other hand, the data highlight the importance of the proper selection of vaccination routes for VTT- or MVTT-based vaccine vectors. Having said so, we do not recommend the use of VTT as a vaccine vector for mucosal vaccination due to its *in vivo* toxicity and pathogenicity [11], [38]. Unexpectedly, we were not able to detect anti-S neutralizing IgA activity (1∶10–1∶20) in the saliva of animals vaccinated with either MVA-S or MVTT-S via any route of vaccination. One possibility is that both MVA-S and MVTT-S have dominantly induced neutralizing IgG responses to block viral transmission and dissemination [11], [38]. We also evaluated supernatants from homogenates of lung and intestines. Due to technical difficulties to exclude Nab contamination from the blood, the results are not presented here. Further careful studies will be needed to determine whether MVTT system would offer advantages for inducing Nab and cell mediated immune responses at mucosal sites. Other viral antigens (e.g. HIV-1) should be also tested to determine whether or not our findings are antigen-dependent.

In conclusion, our replication-competent vaccinia vector MVTT is superior to MVA for inducing high levels of neutralizing antibody via mucosal vaccination. Moreover, the mucosal vaccination may potentially offer significant advantages to overcome the pre-existing anti-VV immunity for people who previously received smallpox vaccination. MVTT is therefore an attractive live viral vector for the development of future mucosal vaccines.
